# Symmetry in Paraspinal Muscles as a Predictor of the Development of Pregnancy‐Related Low Back and Pelvic Pain: A Prospective Study

**DOI:** 10.1111/os.13126

**Published:** 2021-10-19

**Authors:** Gong Long, Zhi‐yuan Fang, Tang Xiang‐sheng, Yang Feng, Ma Hao‐ning, Hao Qing‐ying, Yi Ping, Tan Ming‐sheng

**Affiliations:** ^1^ Department of Orthopaedic China‐Japan Friendship Hospital Beijing China; ^2^ Peking Union Medical College Chinese Academy of Medical College Beijing China; ^3^ Department of Orthopaedic Dongfang Hospital, Beijing University of Chinese Medicine Beijing China

**Keywords:** Asymmetry, Low back pain, Paraspinal muscles, Predictor, Pregnancy

## Abstract

**Objective:**

To determine the asymmetry in the paraspinal muscle before pregnancy and evaluate its association with pregnancy‐associated lumbopelvic pain (LPP).

**Methods:**

This was a prospective case–control study conducted from January 2017 and December 2018. A total of 171 subjects (mean age ± SD, 27.4 ± 5.8 years) were finally divided into the LBP group, PGP group, and no LPP group. Each subject was asked to follow a standardized clinical imaging protocol before the pregnancy. The area of muscles (multifidus, erector spinae, and psoas muscles) on the axial slice at mid‐disc of L_4_–L_5_ and L_5_–S_1_ were segmented and then the cross‐sectional area (CSA) of a particular muscle was measured by outlining the innermost fascial border surrounding each muscle. The mean value of F‐CSA's ratio to T‐CSA (F/T CSA) was used to determine whether the bilateral paraspinal muscle was asymmetrical. Total muscle CSA (T‐CSA) represents the sum of CSA of interested three muscles. The signal intensity can distinguish fat and muscle tissue in a different range. Based on this, functional CSA (F‐CSA), represented by fat‐free area, was evaluated quantitively by excluding the signal of the deposits of intramuscular fat. Total muscle CSA (T‐CSA), functional CSA (F‐CSA), and the ratio of F‐CSA to T‐CSA (F/T CSA) were measured unilaterally and compared between groups. Logistic regression was performed to determine the risk factors for pregnancy‐associated LPP. The Pearson correlation coefficient was performed to test the relationship between asymmetry in F/T‐CSA and pain rating.

**Results:**

A total of 124 subjects (72.5%) (28.5 ± 5.2 years) had LPP during pregnancy. Forty‐eight (38.7%) individuals had low back pain (LBP) and 76 (61.3%) had pelvic girdle pain (PGP). Seventy‐six women (44.4%) were determined to have asymmetry in paraspinal muscle according to the definition in this methods section. The duration of follow‐up was 24 months postpartum. A total of 39 (31.5%) women unrecovered from LPP. F/T‐CSA was significantly decreased for LBP in the PGP group than in the and control group (0.03 ± 0.02 *vs* 0.05 ± 0.03 *vs* 0.12 ± 0.05, *P* < 0.001). Meanwhile, significant differences were detected in both groups (all *P* < 0.001). In patients with LBP, the level of paraspinal asymmetry, represented by the difference in F/T‐CSA, was positively correlated with pain scores (*r* = 0.52, *P* < 0.01). However, no statistically significant correlation between pain scores and paraspinal asymmetry was found in PGP (*r* = 0.42, *P* > 0.05). Asymmetry in the paraspinal muscle (adjusted *OR* = 1.5), LBP (adjusted *OR *= 1.6), LPP in a previous pregnancy (adjusted *OR* = 1.4), sick leave ≥90 days (adjusted *OR* = 1.2), and heavy labor (adjusted *OR* = 1.2) were risk factors for the unrecovered LPP during pregnancy.

**Conclusions:**

Asymmetrical muscular compositions could lead to abnormal biomechanics for the segmental motions. Lateral‐directed physical training and stretching may help decrease the occurrence and severity of this condition.

## Graphical abstract

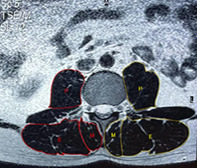

## Introduction

Lumbopelvic pain (LPP) during pregnancy occurs commonly, with a prevalence varying from 20% to 90%, mostly above 50%^1^, ^2^, ^3^. On account of such a significant morbidity, this condition had to be generally considered a natural or trivial problem during pregnancy. Quite a few clinicians are helpless to give a “watch and wait” approach as the best solution for those women. However, severe LPP compromises daily routines such as running and even walking, work performance, and incurs deterioration in the quality of life (QoL) of the patients^4^, ^5^. It is reported that over 80% of pregnant women who experience LBP have limitations in daily activities and 30% of women who suffer severe LPP have to be confined to bed and sick leave from work^6^, ^7^. What's worse, for some women, LPP following childbirth recedes slowly and incompletely, sustaining for several years postpartum, and even becoming a life‐long condition. A total of 51% of women with LPP continue suffering this condition 1 year after delivery^8^, and 20% of women 3 years postpartum^9^. Ostgaard *et al*. have reported that 15% of women experiencing LPP do so after childbearing^10^. The consequences of this high‐incidence disease are pushing those women to seek medical treatment for pain relief, producing economic burdens on individuals and health insurance. These intractable reactions could trigger perinatal depression and sleep disorders, which also threatens the infant health^11^. As a result, LPP ought not to be neglected during pregnancy due to enormous socioeconomic implications and significant influence on the physical and psychological quality of life for the women, their families, and society^12^. Oppositely, these problems are added to the demand for intensive research.

This disease is considered to be clinically complex, including low back pain (LBP) and pelvic girdle pain (PGP)^3^, ^7^. Pain around the symphysis pubis and sacroiliac joints is considered PGP, while LBP is frequently defined as pain between the 12th rib and the gluteal fold^7^. The incidence of PGP is relatively stable during the whole pregnancy, at nearly 10%. By contrast, LBP starts with a low rate in early pregnancy, then climbs and keeps a higher level, about 35% throughout pregnancy^13^. Apart from the frequency, PGP and LBP diminish differently after childbirth. PGP declines to 5% at about 11 weeks postpartum, while LBP does not recede as expected and can even become worse^14^.

Of note, generally, the intensity of both pains worsens as pregnancy progress. This condition is found to be associated with the musculoskeletal system instead of urological or gynecological disorders. Unfortunately, the further pathogenesis and etiology of both LBP and PGP are undefined and multifactorial at present. Changes in posture during pregnancy, weight gain, a shift in the center of gravity, ligament laxity due to pregnancy‐related hormones, fluid retention in connective tissue, and increased intra‐abdominal pressure could contribute to the onset and persistence of LBP^5^, ^15^, ^16^, ^17^. Meanwhile, PGP is considered as a problem which is obviously different from LBP during pregnancy. To be specific, it has been demonstrated that these two pains should be distinguished on account of different etiology and risk factors such as childbearing age, body mass index (BMI), and heavy labor^13^, ^18^.

Numerous studies have demonstrated that exercise, including deep and superficial lumbopelvic muscles, can help reduce the severity of LPP during pregnancy with an improvement in functional ability and physiological adaption^19^, ^20^, ^21^, ^22^, ^23^. This means that paraspinal muscles are of importance to maintain and support the spine's functional stability, and their weakness could participate in the occurrence of LPP^1^. Several clinical studies have indicated that the asymmetry in paraspinal muscles caused by the altered structure, such as atrophy and fat infiltration of the lumbar paraspinal and psoas muscle ipsilateral to the pain side, was observed in patients suffering chronic LPP^24^, ^25^, ^26^. Until now, little is known about whether the same conclusion can be generalized for the LPP in pregnant women. Considering the undefined etiology of this disease, the risk analysis for this problem is significant and worthy of being investigated for the health pregnancy. Therefore, this study's objective was to (i) determine the incidence and characteristics of the phenomenon of asymmetry in the paraspinal muscle on the digital image of magnetic resonance imaging (MRI) before pregnancy; (ii) analyze whether there were associations between LBP and PGP; (iii) to investigate whether asymmetry in the paraspinal muscle was the risk factor for the unsatisfactory recovery from LPP by performing the logistic regression analysis; and (iv) to determine the risk factors for pregnancy‐associated LPP. Multivariate logistic models were performed using stepwise elimination of variables of interest from univariate analysis after adjustment for confounding factors. Discuss the potential clinical treatments that may prevent reducing the risk of pregnancy‐related LPP. We hypothesized that asymmetry in lumbar paraspinal and psoas muscle might be used as a possible predictor for subsequent occurrence of pregnancy‐related LPP.

## Patients and Methods

### Subjects

This was a prospective study from 1 January 2017 to 1 December 2018 where a total of 232 women (mean age ± standard deviation [SD], 26.8 ± 6.3 years) were enrolled in a pre‐pregnancy consultation clinic. This prospective study was approved by the Institutional Review Board (IBR) of the author's hospital, and all volunteers offered a written consent to participate in this research.

Inclusion criteria for the case group included: (i) underwent a 3.0 Tesla MRI scan before pregnancy; (ii) had completed data which could be used for the final evaluation and analysis; and (iii) a prospective study.

Exclusion criteria included: (i) LPP with definite pathology such as lumbar disc herniation, spondylolisthesis, ankylosing spondylitis, infectious diseases, and tumor (N = 21); (ii) a history of spinal fracture, injuries, or back surgery (N = 9); (iii) other chronic diseases, such as hypertension and diabetes mellitus (N = 12); (iv) lumbar muscle training in the last 6 months (N = 6); (v) abnormal pregnancy such as placenta previa, ectopic pregnancy (N = 7); and (vi) lost to follow‐up or incomplete data (N = 6).

Clinical data, including age, BMI, educational background, cesarean delivery, parity, sick leave, heavy labor, and the type of LPP, were collected. According to whether LPP occurred during pregnancy, these women were divided into a case group or a control group.

### Muscle Measurements

Each subject was asked to follow a standardized clinical imaging protocol before the pregnancy. The image was acquired using a 3.0 Tesla MRI scanner (Philips Achieva, Netherlands) with the repetition time (TR) of 2500 ms and echo time (TE) of 60 ms, a matrix of 332 × 266, field‐of‐view of 200 mm, and a slice thickness of 5 mm. The quantitative analysis for the paraspinal muscles on the T2‐weighted axial images was completed using Onis and ImageJ software (Wayne Rasband, National Institutes of Health, Bethesda, MD).

**Fig. 1:**
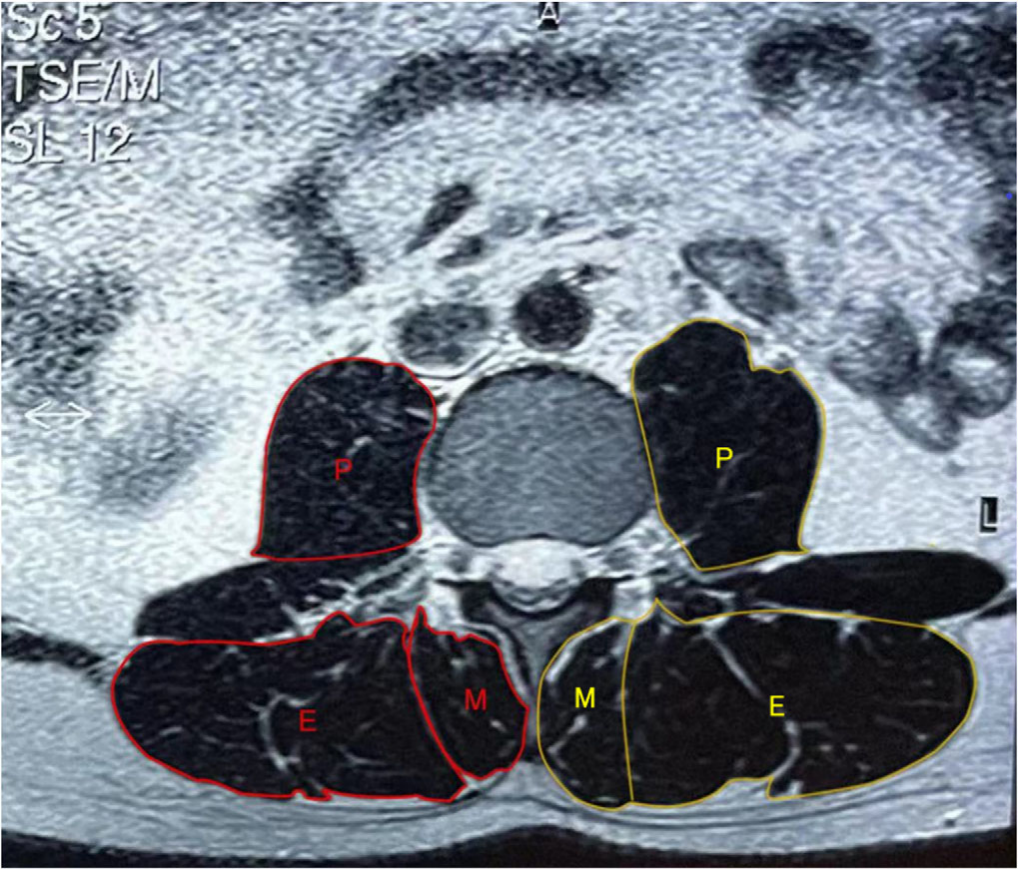
Erector spinae (E), multifidus (M), and psoas muscles (P) were segmented separately on right and left sides on the axial slice at mid‐disc of L_4_–L_5_ and L_5_–S_1_ on T2‐weighted axial images. The above parameters were measured unilaterally. The red line area is functional CSA (F‐CSA), which represents fat‐free area, evaluated quantitively by excluding the signal of the deposits of intramuscular fat. The signal intensity can distinguish fat and muscle tissue in a different range. Based on this, the yellow line area is total CSA (T‐CSA), which represents the sum of CSA of interested three muscles.

One of the authors, who was blinded to the participant's condition, segmented the area of muscles (multifidus, erector spinae, and psoas muscles) on the axial slice at mid‐disc of L_4_–L_5_ and L_5_–S_1_ and then measured the cross‐sectional area (CSA) of a particular muscle by outlining the innermost fascial border surrounding each muscle. Total muscle CSA (T‐CSA) represents the sum of CSA of interested three muscles. The signal intensity can distinguish fat and muscle tissue in a different range. Based on this, functional CSA (F‐CSA), represented by fat‐free area, was evaluated quantitively by excluding the signal of the deposits of intramuscular fat. Then, the ratio of F‐CSA to T‐CSA (F/T CSA) was calculated to determine the extent of fat infiltration of muscle degeneration. The above parameters were measured unilaterally (Fig. 1).

### Lumbopelvic Pain (LPP) and Its Subtypes

Pregnancy‐related LPP ≥3 using the self‐reported scale of 0–10 (0 as no pain to 10 as the worst possible pain) through an interview or telephone call at any appointment during the whole pregnancy was defined as a pain from the lumbar spine or pelvis, lasting for 1 week or longer^14^, as it is frequently considered as a disabling more or less^18^. This rating was served as the primary outcome in this study.

LBP was defined as the pain located above the sacrum in the lumbar spine with a history of lumbar back pain before pregnancy, a limited motion in the lumbar spine, tenderness along with the erector spinae muscle, and negative results on the posterior pelvic pain provocation test (4P‐test).

PGP was defined as the pain located in the gluteal area with no history of lumbar back pain before pregnancy, relation to time‐ and weight‐bearing, pain‐free intervals, an unlimited motion in the lumbar spine, and positive results on the 4P‐test^8^.

Women experiencing LPP were referred to a multidisciplinary team, having an obstetrician, orthopaedist, acupuncturist, and physiotherapist. Arranged treatments, depending on a particular patient's condition, such as pain severity and gestational period^14^, includes patient education, back‐strengthening exercises, rest, physiotherapy, swimming, manipulation, and acupuncture.

### Sample Size

The sample size was 63 patients in each group to detect a mean difference in the pain rating of 0.5 with the SD of 0.25, *α* = 0.05, and 1‐*β* = 0.9, and additional compensation for a dropout rate of 20%.

### Statistical Analysis

We used SPSS version 22 (SPSS; Chicago, IL, USA) to perform all analyses. The mean and standard deviation was used to demonstrate variables, which have been tested to be normally distributed. The chi‐square test was performed to compare ordinal variables, and Student's *t*‐test was used for continuous variables. Then, Tukey's *post hoc* test was used for pairwise comparison. Then patients were divided into asymmetry or symmetry in paraspinal muscle according to F‐CSA/T‐CSA. The cut‐off value of F‐CSA/ T‐CSA was determined by the mean value. Logistic regression was performed to estimate the odds ratio (*OR*) and the associated 95% confidence interval (*CI*) to determine the risk factors for pregnancy‐associated LPP. Multivariate logistic models were performed using stepwise elimination of variables of interest from univariate analysis after adjustment for confounding factors. The Pearson correlation coefficient was performed to test the relationship between asymmetry in F‐CSA/ T‐CSA and pain rating. All measurements were performed by the same doctor. An intraclass correlation coefficient (ICC) was calculated to evaluate intratester reliability with repeated 10 times for all the images. The intra‐rater ICC ranges from 0.92 to 0.98 for T‐CSA and 0.90 to 0.98 for F‐CSA. The intra‐rater ICCs for the between‐sides differences in CSA were 0.82 for erector spinae, 0.87 for multifidus, and 0.90 for psoas. The ICC for the between‐sides difference in the F/T CSA was 0.87 for erector spinae, 0.90 for multifidus, and 0.93 for psoas. The statistical significance and power analysis were *P*‐values ≤0.05 and 0.8, respectively.

## Results

### General Results

A total of 171 subjects (mean age ± SD, 27.4 ± 5.8 years) were included in the final analysis according to the inclusion and exclusion criteria. The duration of follow‐up was 24 months postpartum. A total of 124 subjects (72.5%) (mean age ± SD, 28.5 ± 5.2 years) had the LPP during the pregnancy. Among them, 48 (38.7%) individuals had LBP, and 76 (61.3%) had PGP. After childbirth, 21 (43.8%) women failed to recovery from LBP, and 18 (23.7%) from PGP. Namely, a total of 39 (31.5%) women unrecovered from LPP.

Seventy‐six women (44.4%) were determined as having asymmetry in paraspinal muscle according to the definition in this methods section.

### Comparisons About Demographic Data and Basic Data between Asymmetry and Symmetry in Paraspinal Muscle

As illustrated in Table 1, compared to patients with symmetry in paraspinal muscle, those with asymmetry in paraspinal muscle were more likely to be older (26.4 ± 5.3 *vs* 28.7 ± 5.0, *P* = 0.004), have higher BMI (24.3 ± 2.6 *vs* 26.2 ± 1.8, *P* < 0.001), experience LPP during pregnancy (64.2% *vs* 82.9%, *P* = 0.011), LBP during pregnancy (20.0% *vs* 35.5%, *P* = 0.036), LPP in a previous pregnancy (24.2% *vs* 40.8%, *P* = 0.031), side‐to‐side difference in F‐CSA/ T‐CSA (0.8 ± 0.3 *vs* 1.5 ± 0.6 cm^2^, *P* < 0.001), primigravida (40.0% *vs* 56.6%, *P* = 0.036), sick leave ≥ 90 days (16.8% *vs* 31.6%, *P* = 0.038), and experience heavy labor (17.9% *vs* 39.5%, *P* = 0.003).

**TABLE 1:** Comparisons about demographic data and basic data between asymmetry and symmetry in paraspinal muscle

Variables	Asymmetry in paraspinal muscle (*n* = 76)	Symmetry in paraspinal muscle (*n* = 95)	*P* value
Age (Mean ± SD) (years)	28.7 ± 5.0	26.4 ± 5.3	0.004 ^*^
BMI before pregnancy (Mean ± SD) (kg/m ^2^ )	26.2 ± 1.8	24.3 ± 2.6	<0.001 ^*^
LPP during pregnancy (N, %)	63 (82.9%)	61 (64.2%)	0.011 ^*^
LBP during pregnancy (N, %)	27 (35.5%)	19 (20.0%)	0.036 ^*^
PGP during pregnancy (N, %)	36 (47.4%)	40 (42.1%)	0.594
LPP in a previous pregnancy (N, %)	31 (40.8%)	23 (24.2%)	0.031 ^*^
Side‐to‐side difference in F‐CSA/ T‐CSA (cm2)	1.5 ± 0.6	0.8 ± 0.3	<0.001 ^*^
Smoking (N, %)	9 (11.8%)	11 (11.6%)	0.852
Alcohol user (N, %)	8 (10.5%)	9 (9.5%)	0.977
Educational Levels (≥/Bachelor) (N, %)	45 (59.2%)	51 (53.7%)	0.570
Cesarean delivery (N, %)	12 (15.8%)	16 (16.8%)	0.982
Primigravida (N, %)	43 (56.6%)	38 (40.0%)	0.045 ^*^
Sick leave ≥90 days (N, %)	24 (31.6%)	16 (16.8%)	0.038 ^*^
Heavy labor (N, %)	30 (39.5%)	17 (17.9%)	0.003 ^*^

BMI, body mass index; F‐CSA, functional cross‐sectional area; LBP, low back pain; LPP, lumbopelvic pain; PGP, pelvic gridle pain; T‐CSA, total cross‐sectional area.

However, there were no significant differences in the occurrence of PGP during pregnancy (47.4% *vs* 42.1%, *P* = 0.594) or related to smoking (11.8% *vs* 11.6, *P* = 0.852), being an alcohol user (10.5% *vs* 9.5%, *P* = 0.977), educational levels (≥/Bachelor) (59.2% *vs* 53.7%, *P* = 0.570), and cesarean delivery (15.8% *vs* 16.8%, *P* = 0.982) between patients with symmetry in paraspinal muscle and those with asymmetry in paraspinal muscle.

### Comparisons About Side‐To‐Side Parameters of the Paraspinal Muscles Among Groups

As illustrated in Table 2, in the LBP group, all the mean T‐CSA (18.2 ± 1.7 *vs* 19.6 ± 1.6, *P* < 0.001), F‐CSA (15.5 ± 2.0 *vs* 17.7 ± 1.7, *P* < 0.001) and F‐CSA/T‐CSA (0.72 ± 0.07 *vs* 0.84 ± 0.08, *P* < 0.001) were decreased on the painful or more painful side compared with the no or less pain side. In the PGP group, the difference in mean T‐CSA (18.7 ± 1.9 *vs* 19.0 ± 1.8, *P* = 0.319) between groups was not significant, while those in F‐CSA (16.2 ± 1.6 *vs* 17.0 ± 1.8, *P* = 0.004) and F‐CSA/T‐CSA (0.81 ± 0.06 *vs* 0.86 ± 0.07, *P* < 0.001) on the painful or more painful side were decreased compared with the no or less pain side. However, in those without the LPP during pregnancy, there were no significant differences in the T‐CSA (18.8 ± 1.6 *vs* 19.2 ± 1.7, *P* = 0.243), F‐CSA (17.0 ± 2.0 *vs* 17.3 ± 1.8, *P* = 0.447), and F‐CSA/T‐CSA (0.83 ± 0.15 *vs* 0.86 ± 0.12, *P* = 0.287) between two sides.

**TABLE 2:** Comparisons about side‐to‐side parameters of the paraspinal muscles among groups

Variables	With LPP (*n* = 124)						Without LPP (*n* = 47)		
	LBP (*n* = 48)			PGP (*n* = 76)					
	Pain side	No pain side	*P* value	Pain side	No pain side	*P* value	Left side	Right side	*P* value
T‐CSA (Mean ± SD) (cm^2^)	18.2 ± 1.7	19.6 ± 1.6	<0.001 ^*^	18.7 ± 1.9	19.0 ± 1.8	0.319	18.8 ± 1.6	19.2 ± 1.7	0.243
F‐CSA (Mean ± SD) (cm ^2^ )	15.5 ± 2.0	17.7 ± 1.7	<0.001 ^*^	16.2 ± 1.6	17.0 ± 1.8	0.004 ^*^	17.0 ± 2.0	17.3 ± 1.8	0.447
F‐CSA/T‐CSA (Mean ± SD)	0.72 ± 0.07	0.84 ± 0.08	<0.001 ^*^	0.81 ± 0.06	0.86 ± 0.07	<0.001 ^*^	0.83 ± 0.15	0.86 ± 0.12	0.287

*P* values from ANOVA and *post hoc* analysis.

^*^ Indicates statistically significant.

BMI, body mass index; F‐CSA, functional cross‐sectional area; LBP, low back pain; LPP, lumbopelvic pain; PGP, pelvic girdle pain; SD, standard deviation; T‐CSA, total cross‐sectional area.

### Comparisons About the Ratio of F‐CSA to T‐CSA Among Groups

**Fig. 2:**
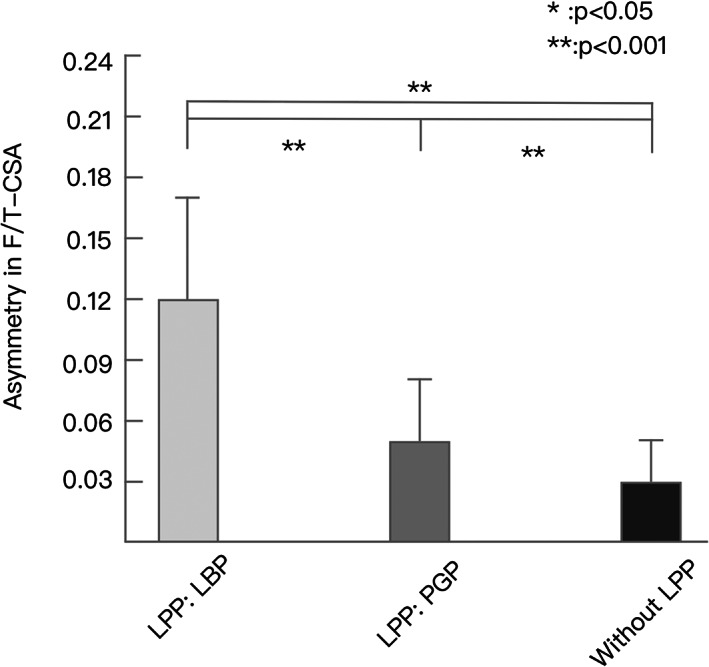
The comparisons about the ratio of functional CSA (F‐CSA) to total CSA (T‐CSA) in low back pain (LBP), pelvic girdle pain (PGP), and no lumbopelvic pain (LPP) group showed that the ratio of F‐CSA to T‐CSA was significantly decreased in LBP than those in the PGP group and control group.

The ratio of F‐CSA to T‐CSA was significantly decreased in LBP than those in the PGP group and control group (0.03 ± 0.02 *vs* 0.05 ± 0.03 *vs* 0.12 ± 0.05, all *P* < 0.001). Meanwhile, significant differences were detected in any two groups (Fig. 2).

### Logistic Regression Analysis for the Unrecovered LPP After the Pregnancy

After adjusting for the remaining covariates, asymmetry in paraspinal muscle (adjusted *OR* = 1.5; 95% *CI*: 1.4–1.6; *P* < 0.01), LBP (adjusted *OR* = 1.6; 95% *CI*: 1.5–1.7; *P* < 0.01), LPP in previous pregnancy (adjusted *OR* = 1.4; 95% *CI*: 1.3–1.5; *P* < 0.01), sick leave ≥ 90 days (adjusted *OR* = 1.2; 95% *CI*: 1.1–1.3; *P* < 0.05), and heavy labor (adjusted *OR* = 1.2; 95% *CI*: 1.1–1.3; *P* < 0.05) were risk factors for the unrecovered LPP during pregnancy. The rest of the variables, including PGP (adjusted *OR* = 1.2; 95% *CI*: 1.1–1.3; *P* = 0.23), BMI ≥ 28 (adjusted *OR* = 1.2; 95% *CI*: 1.1–1.3; *P* = 0.72), smoking (adjusted *OR* = 1.1; 95% *CI*: 1.0–1.2; *P* = 0.26), alcohol use (adjusted *OR* = 1.2; 95% *CI*: 1.1–1.3; *P* = 0.62), educational level (adjusted *OR* = 1.0; 95% *CI*: 0.9–1.1; *P* = 0.29), cesarean delivery (adjusted *OR* = 1.0; 95% *CI*: 0.9–1.1; *P* = 0.33), and primigravida (adjusted *OR* = 1.1; 95% *CI*: 1.0–1.2; *P* = 0.65), failed to be found to predict the development of LPP after adjusting for the remaining covariates (Table 3).

**TABLE 3:** Logistic regression analysis for the unrecovered LPP during pregnancy

Variables	Unrecovered LPP (*n* = 242)			
	*OR*	*P* value	Adjusted *OR* ^†^	*P* value
Asymmetry in paraspinal muscle	1.8 (1.7–1.9)	<0.01 ^*^	1.5 (1.4–1.6)	<0.01 ^*^
LBP types				
No LBP (ref.)				
LBP	2.0 (1.9–2.1)	<0.01 ^*^	1.6 (1.5–1.7)	<0.01 ^*^
PGP	1.5 (1.4–1.6)		1.2 (1.1–1.3)	0.23
BMI≥28	1.6 (1.5–1.7)		1.2 (1.1–1.3)	0.72
LPP in previous pregnancy	1.7 (1.6–1.8)	<0.01 ^*^	1.4 (1.3‐1.5)	<0.01 ^*^
Smoking	1.3 (1.2–1.4)		1.1 (1.0–1.2)	0.26
Alcohol user	1.4 (1.3–1.6)		1.2 (1.1–1.3)	0.62
Educational levels ≥ high school/university	1.2 (1.1–1.3)		1.0 (0.9–1.1)	0.29
Cesarean delivery	1.3 (1.2–1.4)		1.0 (0.9–1.1)	0.33
Primigravida	1.4 (1.3–1.5)		1.1 (1.0–1.2)	0.65
Sick leave ≥ 90 days	1.5 (1.4–1.6)	<0.05 ^*^	1.2 (1.1–1.3)	<0.05 ^*^
Heavy labor	1.6 (1.5–1.7)	<0.05 ^*^	1.2 (1.1–1.3)	<0.05 ^*^

BMI, body mass index; CP, combined pain; LBP, low back pain; LP, lumbar pain; OR, odds ratios; PPP, posterior pelvic pain.

^*^ Indicates statistically significant.

^†^ Fully adjusted by confounding factors. Odds ratios, as well as 95% CI, were showed.

### Linear Regression Analysis between Paraspinal Asymmetry and Pain Ratings

In patients with LBP, the level of paraspinal asymmetry, represented by the difference in F/T‐CSA, was positively correlated with pain scores (*r* = 0.52, *P* < 0.01). However, no statistically significant correlation between pain scores and paraspinal asymmetry was found in PGP (*r* = 0.42, *P >* 0.05).

## Discussion

This study investigated the asymmetry in the paraspinal muscle before pregnancy by measuring the T‐CSA, F‐CSA, and F/T CSA of multifidus, erector spinae, and psoas muscles on the digital image of MRI and evaluated its association with LPP during pregnancy. For these parameters, the measurement was repeatability good, meaning that the present assessments were reliable. Due to the lack of research about the asymmetry in the paraspinal muscle in pregnant women, we compared our results with other similar studies that focused on this condition in other population, such as non‐specific LBP^24^, chronic LBP^25^, ^28^, lumbar disc pathology^27^, and acute LBP^28^. In general, our results are consistent with these studies that there is a reduction of CSA, especially F‐CSA, in the paraspinal muscles of the pain in unilateral LBP or more pain in the bilateral side. Although we found similar results about this data, there were seemingly totally different explanations for pregnant populations and non‐pregnant populations.

### The Possible Mechanism Behind the Occurrence of Muscular Asymmetry in Pregnancy‐Related LPP

The mechanism behind the occurrence of muscular asymmetry is complex and remains unclear at present. First, a decreased CSA of the paraspinal muscles in LBP, whether specific or non‐specific, might arise from the disuse atrophy from the pain stimulation^26^, ^27^, ^28^. Second, the authors assumed that this phenomenon might also result from an inhibition along a long‐loop reflex to protect the pain side's impaired muscles, whether acute or chronic^28^. Third, another study suggested that the side‐related reduction in the paraspinal muscle could be associated with the degenerative changes of the lumbar discs and radiculopathy^29^, ^30^. Namely, nerve root irritation or compression could occur and lead to denervation of the muscle by the nerve root or dorsal ramus injury^31^.

However, the prospective study where the CSA of the muscles was measured on the MRI scans before pregnancy revealed that pregnancy‐related LPP might be closely associated with a reduction in stability due to the asymmetry in paraspinal muscles. Exactly why this is the case for the asymmetry is not known, it could be caused by either pathological or non‐pathological factors such as an imbalance in bilateral muscles around the spine from poor sitting posture. At this stage, the symptoms could be mild or not appear, which does not interfere with daily life. However, the physically adaptive changes, including abdominal and pelvic muscles stretch, uterus expansion, and increased joint laxity, such as the sacroiliac joint and symphysis pubis, significantly affect the spinal biomechanics^32^, ^33^. Under such a condition, the morphological change of the paraspinal muscle, which serves as one of the most important stabilizers^27^, ^28^, could magnify the lumbar instability, and consequently, clinical manifestations as LPP and muscular atrophy appear. There could be a pathologically vicious cycle where the pain leads to muscle spasm and meanwhile inhibits the stabilizing muscles on the painful side, compensatory hypertrophy could occur on the non‐painful side^27^, and these changes further aggravate the imbalance of the paraspinal muscles, making the forces more vulnerable to atrophy.

Our results showed that significant side‐to‐side differences were found in F‐CSA and the ratio of F‐CSA to T‐CSA, but not in T‐CSA. Although parts of individuals have superior T‐CSA of the paraspinal muscles, increased fatty infiltration and less muscular composition exist. This can be reflected more accurately by the lower F‐CSA, especially F‐CSA/T‐CSA. Currently, the etiology of muscular fat filtration is far from clear^28^. Previous research has suggested it is likely multifactorial, such as altered differentiation of the fibroblasts after paraspinal muscle inflammation, age‐related degeneration, and trauma^34^. More muscular contents and less fat naturally have stronger stability for the spine, even when a pregnancy happens. However, the present findings that F‐CSA/T‐CSA was significantly decreased in LBP and PGP than those in the control group further added that asymmetrical muscular compositions could bring out the symptomatic LPP during the pregnancy.

### Logistic Regression Analysis for the Pregnancy‐Related LPP

When it comes to the regression of LPP after the childbirth, logistic regression analysis revealed that LBP (adjusted *OR* = 1.6), LPP in a previous pregnancy (adjusted *OR* = 1.4), sick leave ≥90 days (adjusted *OR* = 1.2), and heavy labor (adjusted *OR* = 1.2) were consistent with several previous studies^7^, ^8^, ^9^, ^10^, ^12^, ^35^. Among these factors, LPP types, LBP or PGP, were considered as a primary reason for the substantially different regression. LBP does not regress as expected, whereas PGP usually diminishes at about week 11 postpartum^14^. Our results showed asymmetry in F‐CSA/T‐CSA is a risk for the unrecovered LPP 2 years after the childbirth (adjusted *OR* = 1.5). OR values in LPP types and asymmetry in paraspinal muscle could reveal similar strengths of the association between this condition and exposures in the case–control study. Another analysis showed that the level of paraspinal asymmetry, represented by the difference in F‐CSA/T‐CSA, was positively correlated with pain scores in LBP instead of PGP^28^. This further confirmed the conclusions in previous studies that LBP and PGP arise from different mechanisms, despite it not being completely clear. Therefore, it is worthy of being postulated that asymmetrical muscles played various parts in the occurrence and development of these two types of pains.

### Clinical Implications for the Present Findings

There are several implications for the present findings. First, the present results may push forward the recognition of the mechanism for this disease. Asymmetrical muscular compositions could lead to abnormal biomechanics for the segmental motions. It is hard to say that a minor change in pathological morphology could not be magnified to incur the appearance of symptoms when a growing uterus happens. However, a biomechanical experiment in a further study is needed to know the whole picture behind these conditions. Besides, higher F‐CSA/T‐CSA, representing an increase in muscular deposition and reduction in fat infiltration, have better muscular contractility to stabilize the spine and thus make these women less likely to experience pain and segmental instability during the pregnancy. Based on this phenomenon, it is seemingly necessary for orthopaedists or obstetricians to arrange MRI scans to screen for asymmetrical muscles for women who have a high risk for developing severe LPP and unsatisfactory regression after childbirth, such as LPP in a previous pregnancy, heavy labor, and depression^10,13,35^. Therefore, morphological parameters, mostly describing the muscle's contents, could be used for predicting the occurrence and development of pregnancy‐associated LPP. It might be helpful for those women to undergo lateral‐directed physical training and stretching to decrease the occurrence and severity of this condition.

### Limitations for the Present Findings

Several limitations exist in our study. First, signal intensity was not measured, so that intramuscular fat filtration was neglected due to the applied methodology. However, the quantitative measurements on other parameters have provided adequate reliability and precision to find the differences between groups instead of qualitative evaluations. Second, a total of three muscles was compared as the whole instead of a particular muscle and that at each segment. Multifidus is reported as the primary respondents to the pains or pathology among previous studies' paraspinal muscles^26^, ^27^, ^28^. Confirming these results needs further study, and it would reveal more detailed information about the mechanism of pregnancy‐related LPP. Third, considering the small sample, we had to control the number of variables in the logistic analysis. This selection bias could decrease the reliability of the present results.

### Conclusion

The ratio of F‐CSA to T‐CSA was significantly decreased in LBP than those in the PGP group and control group. Asymmetry in the paraspinal muscle (adjusted *OR* = 1.5), LBP (adjusted *OR* = 1.6), LPP in a previous pregnancy (adjusted *OR* = 1.4), sick leave ≥ 90 days (adjusted *OR* = 1.2), and heavy labor (adjusted *OR* = 1.2) were risk factors for the unrecovered LPP during pregnancy. These findings may push forward the recognition of the mechanism for this disease. Asymmetrical muscular compositions could lead to abnormal biomechanics for the segmental motions. For those women, it might be helpful to undergo lateral‐directed physical training and stretching to decrease the occurrence and severity of this condition.
